# Neuroimaging of human and non-human animal emotion and affect in the context of social relationships

**DOI:** 10.3389/fnbeh.2022.994504

**Published:** 2022-10-21

**Authors:** Pauline B. Zablocki-Thomas, Forrest D. Rogers, Karen L. Bales

**Affiliations:** ^1^California National Primate Research Center, Davis, CA, United States; ^2^Princeton Neuroscience Institute, Princeton University, Princeton, NJ, United States; ^3^Department of Psychology, University of California, Davis, Davis, CA, United States; ^4^Psychology Graduate Group, University of California, Davis, Davis, CA, United States; ^5^Department of Neurobiology, Physiology, and Behavior, University of California, Davis, Davis, CA, United States

**Keywords:** emotions, affect, imaging, social relationships, neurohormones

## Abstract

Long-term relationships are essential for the psychological wellbeing of humans and many animals. Positive emotions and affective experiences (e.g., romantic or platonic love) seem to be closely related to the creation and maintenance of social bonds. When relationships are threatened or terminated, other emotions generally considered to be negative can arise (e.g., jealousy or loneliness). Because humans and animals share (to varying degrees) common evolutionary histories, researchers have attempted to explain the evolution of affect and emotion through the comparative approach. Now brain imaging techniques allow the comparison of the neurobiological substrates of affective states and emotion in human and animal brains using a common methodology. Here, we review brain imaging studies that feature emotions characterized by the context of social bonding. We compare imaging findings associated with affective and emotional states elicited by similar social situations between humans and animal models. We also highlight the role of key neurohormones (i.e., oxytocin, vasopressin, and dopamine) that jointly support the occurrence of socially contextualized emotions and affect across species. In doing so, we seek to explore and clarify if and how humans and animals might similarly experience social emotion and affect in the context of social relationships.

## Introduction

Social relationships are important for social development and long-term psychological health. These relationships are maintained by behaviors that are tightly linked with various emotions and affective states, which are themselves defined by social and other environmental contexts (Spoor and Kelly, 2004; de Waal, 2011). In the past several decades, the interest in applying functional brain imaging for the study of human emotion has grown considerably (Davidson and Irwin, 1999; Phan et al., 2004; Wager et al., 2008). However, the application of these same methods to study emotion and affect in non-human animals (hereafter animals) is still early in its development.

Recent advances in the field of brain imaging have increasingly allowed for direct observation of neural processes active during presumed emotionally evocative events, particularly in humans, as illustrated throughout this review. In both humans and animals, imaging allows for the relatively non-invasive, longitudinal study of neural processes; the reduced invasiveness of imaging techniques also allows one to study typically non-researched animals (e.g., pets and zoo animals). In particular, positron emission tomography (PET) imaging allows uptake of a radiotracer while an animal is unrestrained, awake, and potentially engaged in ethologically and socially relevant tasks. While imaging does not currently allow us to examine activity at the level of the neuron, it allows for more naturalistic social interactions that other methods (e.g., electrophysiological recording) do not. Another drawback of imaging methods is that they are still indirect measures (e.g., with the use of PET scan, we only measure glucose uptake in the neuron as a proxy for neuron activity) and cannot be used for all behaviors for both animals and humans (e.g., in an fMRI scanner, subjects have to stay immobile, and it also requires to create a stimulus that the subject can easily watch or imagine when laying in the scanner). Despite these limitations, imaging methods are powerful tools and have led to significant advances in the field of behavioral neuroscience. While the study of animals in imaging studies could significantly improve our understanding of the physiological substrates of affective processes, few imaging studies have focused on animals.

Emotion is not easily defined, and several definitions have been put forward (for a recent review, see Paul and Mendl, 2018). The term “affect,” which is a closely related term to emotion, tends to be used interchangeably with the term *emotion* by some authors, although others make a clear distinction between the two terms (Russell, 2003; Shouse, 2005). It has been suggested that an emotion is a multicomponent response (subjective, physiological, neural, and cognitive) to the presentation of a stimulus or event (Paul and Mendl, 2018). Other authors posit that an emotion is, perhaps more broadly, a state of the nervous system that is provoked by extrinsic or intrinsic stimuli (Anderson and Adolphs, 2014). Alternatively, “affect” is often used as an umbrella-term or “superordinate category” to describe, at the most basic level, a number of related constructs including emotions, emotion episodes, mood, dispositional states, and traits, which are themselves largely distinguished from one another according to their durations (Gross, 1998). The concept of affect is also often at the center of the definition of emotional episodes, as a state that is positively or negatively valanced and either activated or deactivated (Russell, 2003; Bliss-Moreau, 2017). We do not seek to debate these definitions, but to promote clarity in this review we offer a classic, broad, functional definition for use in this discussion; i.e., emotions are temporary and relatively brief affective states that result from internal and external stimuli that are situated within social and environmental contexts; and emotions are reflected in changes in the brain and periphery of the body that serve to facilitate a locally rational reaction to those stimuli and motivate action. Emotions are frequently elicited by the expectancy of a reward or a punishment, thus it is also possible to classify emotions into categories of responses to rewarding (i.e., positive) or punishing (i.e., negative) stimuli (Mendl et al., 2010). Additionally, although we define emotions as a relatively brief state, they can occur repeatedly and can also be studied in the long term, for example throughout the duration of social relationships (e.g., in love, or grief), where relationships influence emotion expression for long periods and can have lasting effect on physiology and neurobiology (for example on the dopamine system where attachment is associated with a higher D1 binding in the brain; Hostetler et al., 2017).

A recent review provides a conceptual framework and an exhaustive list of the various methods used to study emotions in animals and further illustrates how complex it is to understand them (Kremer et al., 2020). They suggest that the affective and emotional states of animals can be assessed by four components of an affective episode: (1) The feeling or subjective component is described as a psychological construct that may not be shared by all animals and which is impossible to assess in non-talking animals. (2) The behavioral component is described as a change in behavior that may be a cause or a consequence of emotion depending on the authors (Anderson and Adolphs, 2014) and specific behaviors have been associated with positive and negative emotions and can be quantified to assess the emotional state (positive: e.g., play behavior, anticipatory behaviors, consumptive behaviors, affiliative behaviors; negative: e.g., freezing, aggressive behaviors, displacement behaviors). (3) The cognitive component is described as a source of *cognitive bias* during cognitive tests. It is further described as the result of the bidirectional link between emotional and cognitive processes (judgment, attention, and memory) (Harding et al., 2004; Mendl et al., 2010). (4) The physiological component is described as the observed change in physiological systems (e.g., the neuroendocrine, immune, and autonomic nervous systems) during an affective experience. When assayed individually, any of these four affective components should be interpreted with restraint. When studied in animals, without the potential to directly communicate the psychological component of an affective experience, it may be more difficult to conclude that a change in either behavior, cognition or physiology alone is a result of a shift in affect. Thus, recent studies on animal emotion and affect try to combine several measures of behavior, cognition, and/or physiology; and they often combine multiple components of affect (e.g., physiology *and* behavior) into consideration (Maninger et al., 2017b; Cook et al., 2018; Kato et al., 2018).

Other frameworks exist in contrast to the “classical” view of emotions, for example the “Theories of Constructed Emotion” or TCE (Bliss-Moreau, 2017; Feldman Barrett, 2017), which states, for example, that discrete emotions cannot be consistently attributed to specific brain networks. For example, they would suggest that there is no specific “fear circuit” or “anger circuit.” Rather, the TCE would suggest that discrete emotions are higher order interpretations of more basal and diffuse “affect” that is contextualized within the current social situation. Various elements of affect would themselves be constructed of several interacting brain networks. In this framework, “affect,” when defined as a perturbation in allostasis, is the principal description of emotion, varying across two axes: arousal (high to low) and valence (positive to negative). Affect, combined with context, previously lived experience, and (potentially) the ability to conceptualize, results in emotion. Emotions experienced by animals may or may not be recognizable to humans; but importantly, they did not evolve as modules recognizable by a set of clearly identifiable brain areas or networks if we refer to the TCE. However, in animals as in humans, we are capable of studying biological and behavioral responses to affective stimuli.

Emotions in social relationships, what we will call “social emotions,” are of major importance in animal societies (Spoor and Kelly, 2004). Social emotions are defined by the social context in which they occur (Panksepp, 1998), and which exist as dependent upon the affect, behavior, or cognition of others. Social emotions may be considered adaptive, since they allow for the creation and maintenance of valuable relationships which may subsequently provide fitness benefits (Spoor and Kelly, 2004). Stable relationships are of major importance for animals (including humans) that rely on conspecifics for survival and success, as they provide health benefits and lower mortality risks (House et al., 1988). The frequency of affiliative behaviors that an individual displays with other members of their social group is associated with increased reproductive success in several species (Brent et al., 2014b). Social bonds and associated social emotions may also seem to be accompanied by several “drawbacks,” especially when they are negative in valence, i.e., associated with punishment (distress, pain), as in the cases of loneliness and grief. One might wonder if these negative emotions are adaptive and how they evolved. Here, we posit that negative emotions should not be viewed as “maladaptive” and, to the contrary, may themselves have an adaptive role (Nesse, 1990). Some evidence suggests that during separation, titi monkeys display behavior (such as lip smacking, an affiliative behavior in this species) that may strengthen the valuable relationship (Maninger et al., 2017b). Another (also potentially positive for reproduction) example is seen in jealous macaques, where males react with aggressive behaviors toward their male rival (Rilling et al., 2004), perhaps as an attempt to disrupt the formation of a relationship between his current consort and a potential competitor.

Thus, social emotions seem to be shared across animal species, and there is growing evidence that shared neural and hormonal substrates are involved in the expression of affect and emotion across species as a result of a common evolutionary history (Panksepp, 2011). One approach to understanding animal emotion is through the comparative method (Nesse, 1990), in which human and animal emotional experiences are compared and contrasted. The comparative approach operates on the understanding that because vertebrates share common neural structures, and especially humans with other primates (Hori et al., 2021), they may share the products of those common structures, for example emotions or affective states (Panksepp, 2011). Even if direct comparison between distant species (including birds, fish, and amphibians) is a very delicate operation, some brain areas have been clearly identified as homologous, such as the lateral septum (LS) (Goodson, 2005) periaqueductal gray (PAG) (Kingsbury et al., 2011) and the amygdala (Laberge et al., 2006). Notably, the Social Behavior Network is a good example of remarkably conserved structures across vertebrate species that comprises six “nodes” (or brain regions of interest): the extended medial amygdala, the LS, the preoptic area, the anterior hypothalamus, the ventromedial hypothalamus and the midbrain (Goodson, 2005; O’Connell and Hofmann, 2012). This conserved evolution is also seen in neural ligand and receptors distribution involved in social behaviors such as the nonapeptides of the vasotocin family (including the mammalian oxytocin and vasopressin and their homologs in other taxa), which also present variations shaped by different selection pressures (Goodson, 2008; O’Connell and Hofmann, 2012).

Brain imaging studies have at times revealed robust inter-species variations in neural responses to comparable stimuli between species regarding the same emotions, in macaques and titi monkeys with the use of PET scans for example (Rilling et al., 2004; Maninger et al., 2017b). We suggest that these differences may rely on the diversity of social systems with distinct species not having the same emotional response to similar stimuli, and thus potentially relying on differing neural substrates acquired through evolution (Panksepp, 2011).

In this review, we summarize imaging studies of complex social emotions in humans and animals; here, complex indicates a condition of not falling in the definition of basic emotions (Tracy and Randles, 2011) but rather existing as a construct of several basic emotions (Pedersen et al., 1994; Wang et al., 2020). Moreover, we try to link social emotions with neurohormones and neurotransmitters involved when possible. We then seek to explore how brain imaging might further allow us to study emotions, particularly in animals, and we explore whether animals and humans present similar patterns of brain activity in similar situations, eliciting social emotions as a result of their common evolutionary history. Finally, we conclude that there are striking similarities across species, as well as potential species-specific variation, and we demonstrate how comparative studies also shed light on the importance of the oxytocin network in the neurobiology of social emotions across mammalian species.

In the following article, abbreviations are listed in Table 1.

**TABLE 1:** List of abbreviations.

Type	Abbreviation	Full name
Brain area	ACC	Anterior cingulate cortex
Brain area	BNST	Bed nucleus of the stria terminalis
Brain area	LS	Lateral septum
Brain area	MPOA	Medial preoptic area
Brain area	NAcc	Nucleus accumbens
Brain area	OFC	Orbitofrontal cortex
Brain area	PAG	Periaqueductal central gray
Brain area	PCC	Posterior cingulate cortex
Brain area	PFC	Prefrontal cortex
Brain area	PVN	Paraventricular nucleus
Brain area	PCL	Paracentral lobule
Brain area	SON	Supraoptic nucleus of the hypothalamus
Brain area	STS	Superior temporal sulcus
Brain area	VP	Ventral pallidum
Brain area	VTA	Ventral tegmental area
Brain area	SN	Substantia nigra
Methods	BOLD	Blood-oxygenation-level dependent
Methods	fMRI	Functional magnetic resonance imaging
Methods	PET	Positron emission tomography
Neurotransmitter	AVP	Vasopressin
Neurotransmitter	DA	Dopamine
Neurotransmitter	OT	Oxytocin
Molecule	CRF	Corticotropin-releasing factor
Receptor	CRFR	Corticotropin-releasing factor receptor
Receptor	D1R	Dopamine D1 receptor
Receptor	OXTR	Oxytocin receptors
Receptor	V1aR	AVP receptors
other	NHP	Non-human primates

## Neural substrates of emotional process in social relationships

### Bond creation and maintenance: Maternal love, romantic love, feeling of friendship

#### Romantic and maternal love

We start here with what is perhaps the most famous and most studied social emotion: love. Love has been examined in a variety of contexts, such as that of a mother and her infant (“maternal love”), or between two romantic partners (“romantic love”), which in both cases leads to the formation of an attachment (Ainsworth, 1978). In social species, attachment is often considered a key behavioral process in the life of an individual, especially mammals (Insel and Young, 2001). Romantic and maternal love are linked to reproductive success. Although mother-infant attachment is not necessarily lifelong, and while it is highly dependent on the parental care style of the species (see review: González-Mariscal and Poindron, 2002), the research on mother-infant attachment is interesting for the consideration of the onset of social bonds (Hazan and Shaver, 1987; Numan and Young, 2016). Moreover, it is subserved by similar mechanisms as romantic love as pointed out by a very recent meta-analytic review of functional imaging studies (Shih et al., 2022), suggesting a common evolutionary origin. Indeed, bonding with a primary, parental attachment figure may be important for future attachment with a partner during adulthood (Rogers and Bales, 2020; Savidge and Bales, 2020).

##### Mother-infant attachment

Pharmacological and lesion studies on mother-infant attachment were conducted in animals (Insel and Young, 2001), mostly in rats. These studies implicated the medial preoptic area (MPOA), the bed nucleus of the stria terminalis (BNST), and projection sites such as the lateral habenula and the ventral tegmental area (VTA) (Insel and Young, 2001). As an example, a pharmacological study relied on the experimental manipulation of oxytocin in the VTA and the MPOA and vasopressin in the MPOA, showing how these peptides in these brain regions facilitate the onset of maternal behavior in postpartum rats (Pedersen et al., 1994). For more examples in mammals, see the review of Numan and Young (2016). Also in birds (*Taeniopygia guttata*), there is evidence that the nonapeptide arginine vasotocin (the avian homolog of vasopressin) participates to attachment (search for proximity) between offspring and their parent (Baran et al., 2016). The avian homolog of oxytocin, mesotocin, is involved in the process of imprinting (i.e., attachment to a parental figure), by showing a higher preference for a stuffed hen in newly hatched chicks receiving intracranial mesotocin injections as compared to as vehicle (Loveland et al., 2019).

Therefore, in humans, researchers specifically examined regions rich in oxytocin (OT) and vasopressin (AVP) receptors (Loup et al., 1991; Freeman and Young, 2016) with fMRI when studying mother-infant attachment (Lorberbaum et al., 2002; Bartels and Zeki, 2004). Researchers utilized fMRI in humans to observe the brain activity of mothers while they looked at pictures of human faces, including their own infant, familiar children (a first control condition), and their best friend and other familiar adults (as additional controls). They first described striking similarities between maternal love and romantic love (from their previous study: Bartels and Zeki, 2000). Bartels and Zeki (2004) found that the induction of maternal and romantic love both activated regions that belong to the reward system (Bartels and Zeki, 2004), some of which may contain a significant density of oxytocin and vasopressin receptors in humans (e.g., substantia nigra for OTR and AVPR1a, and the globus pallidus and the ventral pallidum for OTR only; Loup et al., 1991; Frehner et al., 2022). Both types of love also activated the brain regions involved in the reward system (therefore implicating dopamine), as well as decreased activation of brain regions related to social judgment and regions associated with negative affect (Table 2). Maternal love also activated specific areas that were not active during romantic love, such as the PAG, a region with oxytocin and vasopressin receptors, as well as the lateral orbitofrontal cortex (OFC) (Bartels and Zeki, 2004; Noriuchi et al., 2008). The involvement of the PAG in maternal love was subsequently confirmed by another fMRI study (Noriuchi et al., 2008).

**TABLE 2:** Summary of the brain areas involved in the expression of “love” across species.

Species (Sex)	How to elicit it (*Imaging method*)	Neural changes (↗increase/↘ decrease)	References
Love and attachment maternal and romantic			
Titi monkey–*Plecturocebus cupreus* *(Males)*	Males involved in long-term pair bond as compared to newly paired males or lone males *(PET scan)*	*Pair-bonding* **↘ nucleus accumbens, ventral pallidum**, medial preoptic area, medial amygdala, and the supraoptic nucleus of the hypothalamus, lateral septum	Bales et al., 2007
	Before and 48 h after pair-bonding *(PET scan)*	↘ right **nucleus accumbens** and ventral pallidum	
Titi monkey–*Plecturocebus cupreus* *(Males)*	Newly paired males (1 week) and long-term paired (4 month) as compared to non-paired males *(PET scan)*	↗ **nucleus accumbens, ventral pallidum, caudate, putamen** **(“motivational areas”)**	Maninger et al., 2017a
Titi monkey–*Plecturocebus cupreus* *(Males)*	Newly paired males (4–8 weeks after pair formation) compared to unpaired males *(PET scan)*	↗ lateral septum (D1R binding)	Hostetler et al., 2017
Sprague Dawley Rats–*Rattus norvegicus* (Females)	Pup stimulation *via* suckling *(fMRI)*	*Mother-infant attachment* ↗ **nucleus accumbens, caudate nucleus**, **putamen**, prefrontal cortex **(“reward pathway”)**	Ferris et al., 2005
Humans–*Homo sapiens* *(Men and Women)*	Deeply involved adults scan presented to the picture of the loved one compared to the picture of a friend *(fMRI)*	*Romantic love* ↗ medial insula, **anterior cingulate cortex**, **caudate nucleus** and the **putamen** ↘ amygdala, posterior cingulate gyrus, right prefrontal, parietal and middle temporal cortices	Bartels and Zeki, 2000
Humans–*Homo sapiens*	Mothers presented to pictures of their own compared to a picture of acquainted children, of their best friend and of acquainted adults *(fMRI)*	*Specific to Maternal love* ↗ periaqueductal central gray, lateral orbitofrontal cortex *Common to Romantic and maternal love* ↗ medial insula, **anterior cingulate cortex; caudate nucleus**, **putamen**, the globus pallidus, ventral tegmental area, substantia nigra ↘ amygdala, posterior cingulate cortex, middle prefrontal, inferior parietal, middle temporal cortices, temporal poles, parietotemporal junction, mesial prefrontal cortex	Bartels and Zeki, 2004
Humans–*Homo sapiens* *(Men and Women)*	Participants intensely in love watching picture of their beloved partner compared to acquaintance. *(fMRI)*	↗ ventral tegmental area, **caudate nucleus** *But also for longer-term relationships:* **mid-insular cortex, right anterior** and posterior **cingulate cortex**	Fisher et al., 2005
Humans–*Homo sapiens*	Mother watching videoclips of their child compared to other children. *(fMRI)*	*Maternal love:* ↗ periaqueductal central gray orbitofrontal cortex, anterior insula, and dorsal and ventrolateral parts of putamen.	Noriuchi et al., 2008
Humans–*Homo sapiens* *(Men and Women)*	Comparison of long-term couples to long-term friendship or familiar neutral *(fMRI)*	↗ **nucleus accumbens**, **ventral tegmental area**, **caudate nucleus**, **cerebellum**, **putamen**, mid-insula, posterior hippocampus, left, globus pallidus, substantia nigra, raphe nucleus, thalamus, anterior cingulate cortex, posterior cingulate cortex, periaqueductal gray, hypothalamus + *increase of nucleus accumbens activation over the years*	Acevedo et al., 2012
Humans–*Homo sapiens* *(Men and Women)*	Newly married couple resented to a picture of partner or neutral acquaintance (highly familiar) at two time points: around the weeding date and approximately 11 months after the wedding	↗ substantia nigra, left paracentral lobule (PCL); ventral tegmental area, insular cortex, periaqueductal gray, posterior hippocampus, occipital cortex, Septum/fornix region ↘ anterior temporal gyrus, inferior frontal gyrus, Superior temporal gyrus/angular gyrus	Acevedo et al., 2020
Humans *Homo sapiens* *(Men)*	Pair-bonded male volunteers presented to pictures of their bond or another women *(fMRI)*	↗ **nucleus accumbens**	Scheele et al., 2013
Humans–*Homo sapiens* *(Men and Women)*	Participants involved in a romantic relationship and very intensely in love, presented to pictures of their partner or a familiar acquaintance *(fMRI)*	↗ **ventral tegmental area**, **caudate nucleus** (tail), **cerebellum**, middle orbitofrontal cortex ↘ right amygdala, right **accumbens**, right medial orbitofrontal	Xu et al., 2011

Names in bold are brain areas regularly found in neural changes related to emotion.

Comparing maternal love and related experiences of mother-infant attachment or affiliation in humans and rodents is challenging; however, one scenario where this emotion could presumably be expressed is when mothers are directly interacting with their pups. In rats, it is possible to induce the onset of maternal behaviors by repeated exposure to pups (Fleming and Rosenblatt, 1974). The neural activations are similar to that observed during episodes of human maternal love has been found using fMRI in nursing female rats. The suckling of pups activated the dopamine reward system, which may help reinforce the bond between the mother and the pup (Ferris et al., 2005). In spite of the variable sensory conditions between rodent and human studies (for instance, use of tactile stimuli in rats vs. psychological stimuli in human), neural activity converges on a common pathway, which is thought to involve reward. Infant cry studies in humans simulated separation of the mother from her infant, and were also conducted to study mother-infant attachment. One of the first fMRI studies using infant cries to study mother attachment compared brain activations of mothers listening to their baby cries and white noise (Lorberbaum et al., 1999, 2002). The second study confirmed the implication of the BNST, the MPOA and the VTA in the maternal response to distress call, as well as the anterior cingulate cortex (ACC). The ACC has been implicated in both the neurobiology of the distress call from the infant, and in the response of the mother (see more in the section “Separation from a valuable relationship: Social pain, loneliness, and grief”).

##### Pair mate attachment and romantic love

Definitions of a pair bond across species generally require a selective association between two adults that contains an affective component and lasts outside of one reproductive cycle (Bales et al., 2021). Pair bonds as we understand them in mammals are latent psychological constructs that cannot be directly assessed, but which are operationalized by the presence of particular behaviors (e.g., maintenance of close physical proximity) in combination with an emotional component (e.g., attraction or arousal) (Bales et al., 2021).

As mentioned above, human volunteers that felt deeply in love were imaged with fMRI while observing a picture of their romantic partner (compared to a friend) (Bartels and Zeki, 2000). Men and women presented the same activation pattern in the brain, and surprisingly, the authors described a limited number of areas that were activated: increased activity in the medial insula, in the ACC, and the caudate nucleus and the putamen, all bilaterally. In addition, deactivations (a decrease of the BOLD signal as compared to the control condition or negative BOLD response) were observed in the posterior cingulate gyrus, in the amygdala and were right-lateralized in the prefrontal, parietal and middle temporal cortices. These results are supported by their second study (Bartels and Zeki, 2004) focusing on brain regions known to contain high densities of oxytocin and vasopressin receptors (Loup et al., 1991). Deactivations were similar to the previous study (see also Table 2; Bartels and Zeki, 2004). They found that the VTA, the dentate gyrus/hippocampus and the hypothalamus were only activated by romantic love, which was not previously detected. Indeed, a following study on intense romantic love also found that the VTA (and the caudate nucleus) were very important in the formation of romantic bonds, by implicating the reward system (Fisher et al., 2005). This later cited study also points out a very important finding: they report that in couples with longer relationships (8–17 months as compared to new relationships), other brain regions had a higher BOLD signal, including the right mid-insular cortex, the right ACC and PCC, and the right posterior cingulate/retrosplenial cortex. As such, this suggests that the brain areas involved in romantic relationships evolve through time rather quickly. Four other fMRI studies on passionate love were included in a meta-analysis and they were also consistent with the finding that love was recruiting areas linked with reward and emotions, but also social cognition, attention and self-recognition (Ortigue et al., 2010).

Presentation of a picture of a romantic partner, compared to a friend or highly familiar acquaintance, can be used to study the various aspects of love. For example, a group of Chinese participants was imaged using fMRI with the intention of detecting any divergences between Western and Eastern cultures (Xu et al., 2011). Indeed, it is problematic that most human studies cited above and below had been conducted on WEIRD participants (Western, Educated, Industrialized, Rich, and Democratic) or even STRANGELY WEIRD (people who interact with Social media, engage in Temporary relationships, can Relocate with relative ease, have Autonomy in mate choice, are Nulliparous, experience social Group segmentation, are being tested in an Educational setting, have Lots of options, and are Young adults) in this area (Henrich et al., 2010; Goetz et al., 2019). The Chinese participants also presented brain activation in dopamine-rich regions and mid-OFC, as well as a deactivation of the amygdala, the medial OFC and the medial accumbens. This work suggests that human culture does not affect the neural response in romantic love and is a reminder that diversity in the origins of participants should be considered more often in human studies. However, as noted by the authors (Xu et al., 2011), the finding that medial OFC and the medial accumbens activity is decreased in the early stage of love conflicts with other findings in love, maternal love and friendship (Bartels and Zeki, 2004; Acevedo et al., 2012; Azzi et al., 2012; Watson and Platt, 2012; Brent et al., 2014a). The authors argue that the intensity and the direction of the fMRI activation/deactivation in a particular brain area is less relevant than the detection of a change as compared to a control condition.

A more recent study in humans also investigated which brain regions were associated with love maintenance (i.e., Eros change, defined as the difference of general couple satisfaction between the two time points) over the first year of bonding by identifying which parts were activated at the onset of a marriage and a year after (Acevedo et al., 2020). Love maintenance was associated with activation (increase of the BOLD signal) of a dopamine rich region, the SN and with the paracentral lobule (PCL) at the two time points, and with deactivation on the inferior frontal gyrus. This study confirmed the role of dopamine rich regions, and also the importance of oxytocin and vasopressin receptors in brain expression in a love maintenance context, as they also provide evidence of the interaction of vasopressin (AVPR1a rs3), oxytocin (OXTR rs53576), and dopamine (DRD4-7R) receptor gene variants with the activation of the VTA during the feeling of love. To our knowledge, there is currently no evidence of OXTR binding in the VTA in primates, while it exists in rodents (Peris et al., 2017). Finally, the role of dopamine in romantic love in humans was also confirmed by a study using ^[11C]^raclopride, a dopamine D2/D3 receptor antagonist (Takahashi et al., 2015).

Most research on the neural substrates of pair bond formation, neuroimaging studies and otherwise, has been performed in rodents and humans. Here, we have primarily described neuroimaging studies in humans. While pair bonding is rare among non-human primates (NHP), a few species share this social organization with humans (Kleiman, 1977; Fuentes, 1998; Bales et al., 2021). Significant work examining the neural substrates of pair bond formation has also been performed in monogamous NHP, like titi monkeys, with the use of PET scans co-registered with MRI. In brief, conducting a PET scan on an NHP consist in injecting a radio-tracer (F^18^-glucose or any compound of interest) to the subject before a behavioral experiment during the radiotracer uptake period, where the subject is fully awake and unrestrained. After the uptake period, the subject is anesthetized and scanned to identify which brain regions had a higher radiotracer uptake during the behavioral experiment. For example, radiotracer uptake in male titi monkeys was compared before and 48-h after pairing with a female mate. The authors found differing glucose uptake in long-term pair bonded males than in lone males in specific brain areas related to bonding (Nacc, VP, MPOA, Amyg, SON, and LS), indicating sustained differences in neural activity in those areas (Bales et al., 2007). More recently, in a study of pair bond formation in male titi monkeys, patterns of glucose uptake in short-term pair-bonding in PET scans were similar to brain areas commonly found in humans and rodents with other methods, specifically in the dopaminergic and motivational areas, as well as areas involved in social cognition (Maninger et al., 2017a).

##### Long-term attachment

How can love last a lifetime? Is love at its onset the same as after several years of bonding (Eastwick et al., 2019)? In a study of long-term attachment in humans (people married an average of 21.4 years), neural activation through fMRI showed many similarities with shorter-term studies, including increased activation in the reward system in response to images of romantic partners (Acevedo et al., 2012). Reported feelings of love correlated with the activation of the VTA and the caudate nucleus, while feelings of friendship correlated with a globus pallidus (GP) response. Interestingly, the left amygdala was active for long-term romantic love while it is deactivated for early stages of romantic love. In addition, a greater activation of the NAcc and the caudate were associated with the number of years of marriage and sex frequency with their partner (Acevedo et al., 2012).

Neural patterns associated with love in early stages of romantic relationships may predict the maintenance of feelings of love as relationships age. A follow-up experiment re-assessed the experience of love 40 months after an initial fMRI study on 18 participants; researchers investigated whether the neural patterns associated with a love-related stimulus at early stages of a relationship was predictive of later relationship happiness (Xu et al., 2012). The activity of certain brain areas (i.e., anterior medial OFC, right subcallosal cingulate, and right accumbens) at early stages of the relationship were negatively correlated with the scores of relationship happiness 40 months later, while there was a positive relationship between happiness 40 months later and the activity of the caudate tail and posterior medial OFC, implicating them in the maintenance of happiness in long-term relationships (Xu et al., 2012). However, this study did not conduct a second fMRI investigation at the second timespoints 40 months later in order to see whether these brain regions were still implicated at the later stage of the relationship.

In titi monkeys, twelve long-term paired males (i.e., around 1 year of pairing) and five unpaired males were compared using PET scans in order to determine which brain regions may be involved in the maintenance of long-term relationships (Bales et al., 2007): surprisingly, males in long-term pair-bonds had significantly lower relative uptake in the NAcc, VP, MPOA, medial amygdala, supraoptic nucleus of the hypothalamus (SON), and LS than lone males. The lone males were subsequently paired and scanned after 48 h. As previously described in regard to short-term bonding, increases in glucose uptake were observed both globally and regionally in motivational and social memory areas following 1 week of pairing (Maninger et al., 2017a). These results observed in long-term bonding may seem to contradict those of the previously cited study (Bales et al., 2007). However, the issue is a methodological one. In the 2007 study, regional glucose uptake was normalized by dividing by whole brain uptake. However, the subsequent study found that whole brain uptake itself was up-regulated with pairing (Maninger et al., 2017a). When regional uptake was adjusted instead by injected dose of radiation, dopaminergic and social memory areas showed increases over and above those shown in whole brain glucose uptake.

Dopamine is also implicated in the long-term maintenance of pair bonds in titi monkeys. In one study, researchers used a dopamine receptor antagonist (D1) marked with a C^11^ radiotracer to visualize and compare (*via* PET scan) D1 binding before pair bond formation and then 4–9 weeks (i.e., a long-term pairing) after pair bond formation in titi monkeys (Hostetler et al., 2017). Long-term pairing was associated with an increase in D1 binding, specifically in the LS. This particular finding is notable because in titi monkeys, the LS also has oxytocin receptors (Freeman et al., 2014b).

Imaging studies across species seem to converge to very similar findings about love and attachment, in humans, NHP, and rodents, implicating motivational areas involving dopamine and social areas involving oxytocin (Table 2), therefore supporting the existence of a “socially rewarding mechanism” underlying love (or similar affective states in non-humans) and attachment (Preston, 2017). Several moderating aspects have been identified, including effects of culture, relationship duration, and relationship type (e.g., maternal-offspring or mate-mate attachment). In contrast to the investigation of maternal and romantic love, there is a complementary line of research examining platonic love. Thus, while less researched, there is a body of literature that examines feelings and responses associated with friendship.

#### Emotions and affect associated with friendship

Friends are often used as control subjects in studies of love. The complex set of emotions expressed during a social situation involving a friend or a close social bond is something we refer to as a “feeling of friendship.” In animals, friendship is often referred to as a social bond, and the definition of a social bond is based on the quality of the relationship and the pattern of interactions between the two individuals: friends engage in bidirectional affiliative (non-aggressive, non-reproductive) interactions at a higher rate and more consistently than non-friends (Brent et al., 2014a).

In human studies friends are often used as controls for studies focused on romantic love, although some studies have focused explicitly on friendship. In an fMRI study, brain activation elicited by friends was compared with that elicited by neutral family acquaintances (i.e., relatives that were variably familiar to participants, Acevedo et al., 2012). Activation of the posterior GP and the insula was identified during the feeling of friendship (Acevedo et al., 2012). Moreover, authors identified common activation in romantic love and platonic love in the medial OFC, the hypothalamus, the PAG and also in the left hemisphere of the cerebellum. In another human study (Güroğlu et al., 2008), the experience of friendship was not considered a social emotion but rather as an important experience that activates similar brain areas involved in empathy and reward expectancy. The authors compared the neural activity between three types of relationships (positive, negative, and neutral) and three types of items (peers, celebrities, and objects). They found higher activation in the amygdala and hippocampus, the NAcc, and the ventro-medial PFC when subjects interacted with their friends (i.e., positive peers) than with other peers and celebrities.

In a previous review on the neuroethology of friendship (Brent et al., 2014a), authors have suggested that the OFC in humans and NHPs plays an important role for social behaviors and bonding, and that social interaction may be intrinsically rewarding. For example, single neuron recording in the OFC indicated an activation associated with social cues (Azzi et al., 2012; Watson and Platt, 2012) and motivational rewards in macaques. In another study in rhesus macaques, the ACC neuron activity was associated to reward allocation to another individual (Chang et al., 2013). However, to date, we have been unable to identify published references for whole brain imaging of non-human animal friendships.

Although friendship is seemingly less investigated than maternal or romantic love, the patterns of neural activation associated with friendship similarly imply activation of the reward system (see Table 3). In addition, animal models are particularly interesting for the study of friendship, as closeness can be similarly evaluated through social behavior in humans and animals. Friendship is also of interest because of its connection to the phenomenon of trust (Fareri et al., 2020). Notwithstanding that few studies have studied the neural substrates of the feeling of friendship, rewarding experiences and related brain activations are nevertheless increased in presence of a friend or a close social relationship (Fareri et al., 2020). Similar brain regions are activated when two friends are experiencing the same event (Parkinson et al., 2018), demonstrating some level of neural and affective synchrony between friends and potentially the importance of social relationships in emotional processes. There is now additional compelling evidence of the importance of friendship for health (Dunbar, 2018). Relatedly, it is important to have a thorough understanding of the affective experiences of love and friendship under positive circumstances in order to then explore what may occur when important social bonds are disrupted or threatened.

**TABLE 3:** Summary of the brain areas involved in the expression of the *Feeling of* friendship across species.

Species (Sex)	How to elicit it (*Imaging method*)	Neural changes (↗increase/↘ decrease)	References
*Feeling of* Friendship			
Macaques–*Macaca mulatta* *(Males)*	Individuals presented to a rewarded task in the presence of social vs. non-social stimulus *(single neuron imaging)*	↗ **orbitofrontal cortex**, anterior cingulate cortex	Azzi et al., 2012; Watson and Platt, 2012; Chang et al., 2013
Humans–*Homo sapiens* *(Men and Women)*	Long-term friendship compared to neutral relationships *(fMRI)*	*Feeling of Friendship* ↗ posterior globus pallidus, insula *Common to friendship and love* ↗ medial **orbitofrontal cortex**, hypothalamus, periaqueductal gray, left cerebellum	Acevedo et al., 2012
Humans–*Homo sapiens* *(Men and Women)*	Friendship (positive peer) compared to non-relationship (celebrities) *(fMRI)*	↗ amygdala, hippocampus, nucleus accumbens, ventro-medial prefrontal cortex	Güroğlu et al., 2008

Names in bold are brain areas regularly found in neural changes related to emotion.

### Threat to a valuable relationship: Jealousy

Jealousy is an emotional response to a perceived threat to a valuable relationship, and it is a complex emotion that is generally characterized as constructed of several basic emotions, such as fear of loss, anxiety, suspiciousness, and anger about betrayal (Cubicciotti and Mason, 1978; Parrott and Smith, 1993). The response to the threat can elicit various behaviors, such as proximity seeking or aggressive behaviors depending on sex and species (as we will see below). For example, jealousy may be associated with violence toward the partner in men but also in women (de Weerth and Kalma, 1993; Harris, 2003). The jealousy response in humans can be associated with psychological factors such as self-esteem and how much the relationship is threatened (Sharpsteen, 1995) as well as emotional dependency (Buunk, 1982).

In humans and animals, it is possible to create social situations that should elicit jealousy and to observe behaviors or self-reported affective experience that indicate the elicitation of jealousy. While it is still easy in humans to confirm the induction of jealousy with self-report measures (Takahashi et al., 2006; Steis et al., 2021), studying jealousy in animals requires a specific stimulus situation intended to jeopardize the valuable relationship (Winslow et al., 1993; Rilling et al., 2004; Maninger et al., 2017b; Cook et al., 2018; Webb et al., 2020). A jealousy scenario is, for example, a situation in which a potential new bond could be formed between a third individual (or stranger) and one of two members of an established pair-bond. One advantage of using animal models here is the possibility to simulate more realistically a situation where the bond is in danger while still maintaining some control over the consequences. Typically, in human studies, jealousy-inducing scenarios (or vignettes) are proposed by the experimenter to participants so that the participants can imagine themselves in said jealousy-inducing situation; or alternatively, participants are engaged in a *Cyberball* game where they experience social exclusion during a game (Zheng et al., 2021). During this game, participants virtually exchange a ball between two other (fake) players– the participants are led to believe that the other players are real people, when in fact participants are playing with a computer. At some point, the other players stop playing with the participant, thus simulating social exclusion. In the studies presented below, the protocols were specifically designed to elicit and study jealous reactions in humans and animals, and they provide a clear mention of this goal.

In humans, two types of jealousy have been defined in the contexts of social relationships with a romantic partner: sexual jealousy, involving sexual infidelity; and emotional jealousy, involving a loss of investment from the partner in the relationship (Buss et al., 1992; Buss and Haselton, 2005). It is also commonly alleged that men are more prone to sexual jealousy and women to emotional jealousy (Buss et al., 1992), which can be explained from an evolutionary perspective: sexual infidelity carried out by the female partner could lead the male partner to invest in paternal care for an infant/child that is not his own; and emotional infidelity on the part of the man could lead to a decreased investment in existing offspring of the couple. This explanation is notably heteronormative, and it may overemphasize maternal reliance on or concern about paternal care in a cooperatively breeding context in which alloparental care is potentially available. Nevertheless, these two “jealousy-types” have been distinguished in the human brain using fMRI (Takahashi et al., 2006), such that both types of jealousy were investigated in men and women responding to a questionnaire and undergoing an fMRI while thinking about scenarios of infidelity. While self-report measures indicated that men and women felt equally jealous for both types of infidelity (i.e., both sexual and emotional), brain imaging revealed distinct patterns of brain activation between sexes (Takahashi et al., 2006). In women, the posterior STS showed an increased activation correlated with the rating on emotional jealousy, and other cortical regions and the thalamus. The posterior STS is commonly involved in interpretation, deception, trustworthiness, and violation of social norms (Winston et al., 2002). However in men, insula, cortex and thalamus activation were detected and correlated to the rating on emotional jealousy. The amygdala and the hypothalamus (implicated in appraisal of sexual salience and reproductive behavior) were also implicated in male sexual jealousy as shown by the increase in BOLD signal in the study of Takahashi et al. (2006). Both men and women showed an activation of the visual cortex while thinking about sexual infidelity and of the visual cortex and the thalamus while thinking about emotional infidelity. This study is an important reminder of potential sex effects in the neural bases of emotion, even when self-report does not appear to differ by sex and/or gender. In addition, a more recent fMRI study focusing on women that suffered from sexual infidelity by their romantic partner presented a larger spectrum of brain activations when listening to the description of their experience than in the previous cited study (insula, ACC, PCC, mPFC, substantia nigra, GP, nucleus subthalamicus, and hypothalamus). It is interesting to note that in this more “ecologically” relevant scenario (with an actual experience of sexual infidelity), the pattern of activation is not only larger, but also includes regions involved in men jealousy (thalamus) and male monkeys (insula, PCC, and ACC), showing the importance of the experimental design to elicit the desired emotional responses in human subjects.

To date, we have identified only two other imaging studies on the neural basis of jealousy in NHP (Rilling et al., 2004; Maninger et al., 2017b) and one in dogs (Cook et al., 2018). In male rhesus monkeys, researchers elicited a behavioral reaction in individuals seeing their consort, a receptive female from his social group, with another male in the jealousy condition or alone in the control condition. Notably, while not characterized as socially monogamous in contrast to other NHP like titi monkeys, macaques still form temporary social relationships during the estrus of the female. In male titi monkeys, subjects viewed a stranger male in proximity to a stranger female in the control condition, and a stranger male in proximity to the subject’s pair mate (Maninger et al., 2017b). Behaviorally, macaques presented more aggressive behaviors during the jealousy condition without a significant increase or decrease in stress or affiliative behaviors. In contrast, titi monkeys displayed more lip-smacking behavior (an affiliative behavior) in the jealousy condition and showed higher plasma cortisol and testosterone. In a PET scan following the presentation of a jealousy/control situation, macaques that responded more intensely to the jealously inducing stimulus presented right activation of the STS and the right amygdala, the right cerebellum, and a bilateral activation of the insula. While the right hemisphere is often associated with negative stimulus in humans, the STS, and the insula are associated with increased vigilance, face-processing and judgments of trustworthiness. In titi monkeys, PET scan imaging revealed a higher activation of the right LS, the left PCC and the left ACC, and interestingly, a decrease in the right amygdala, during the jealousy condition. This contrasts with the study in male macaques (Rilling et al., 2004) and in jealousy in men (Takahashi et al., 2006), both of which found an increase in right amygdalar activation.

Patterns of brain activation associated with jealousy in male macaques are comparable to patterns identified in human women (Takahashi et al., 2006): namely the activation of the right STS, which was related to increased vigilance in humans (see Table 4). They were also comparable to human men in the activation of the right amygdala. The insula, which has been associated in humans with the judgment of trustworthiness and perception of visceral responses to emotional stimuli, was activated in jealous male macaques but not in jealous humans. During the control condition, male macaques showed a greater relative activation in the thalamus, left precentral gyrus, left cuneus, left amygdala, and right cingulate sulcus. The authors interpret the left activation of the amygdala as a positive attraction to the female. Interestingly, this particular activation of the left amygdala during the control condition was correlated with the activation of the right amygdala during jealousy. The activation of the amygdala can be associated with jealousy in humans, macaques and dogs, and can be related to aggressive behavior (Cook et al., 2018). In dogs kept still in an MRI scanner, subjects in a jealousy-inducing condition reacted with higher activation of the amygdala when witnessing their caregiver giving food to a fake dog, in contrast to dogs in a control condition in which their caregiver simply placed food in a bowl (Cook et al., 2018). Notably, the authors of this study warn that the amygdala activation should not be automatically associated to only one specific emotion, but rather to a higher state of arousal.

**TABLE 4:** Summary of the brain areas involved in the expression of jealousy across species.

Species (Sex)	How to elicit it (*Imaging method*)	Neural changes (↗ increase/↘ decrease)	References
Jealousy			
Humans–*Homo sapiens* *(Men and Women)*	Self -imagination of an infidelity event *(fMRI)*	↗ women: posterior STS; thalamus and cortical regions ↗ men: **amygdala**; hypothalamus	Takahashi et al., 2006
Humans–*Homo sapiens* *(Women)*	Listening to an experienced event of sexual infidelity *(fMRI)*	↗ insula, ACC, PCC, mPFC, substantia nigra, globus pallidus, nucleus subthalamicus, and hypothalamus	Steis et al., 2021
Titi monkeys–*Plecturocebus cupreus* *(Males)*	Presentation of a stranger male next to the female pair bond *(PET scan)*	↗ right lateral septum, left posterior cingulate cortex ↘ right medial **amygdala**	Maninger et al., 2017b
Rhesus macaques–*Macaca mulatta (Males)*	Presentation of a stranger male next to the consort *(PET scan)*	↗ right STS and right **amygdala**; bilateral insula	Rilling et al., 2004
Dogs–*Canis familiaris* (sex not mentioned)	Presentation of the owner giving food to a plastic dog or a bowl *(fMRI)*	↗ **amygdala** correlated with the aggressive temperament	Cook et al., 2018

Names in bold are brain areas regularly found in neural changes related to emotion.

It is necessary to note that there is a significant difference in the methodology used in the respective, aforementioned studies in macaques and titi monkeys: i.e., the titi monkeys were shown a stranger male in both conditions whereas the macaques observed a stranger male only in the jealousy condition (Rilling et al., 2004; Maninger et al., 2017b). One interpretation could be that the presence of a stranger male could activate the right amygdala in macaques in the jealousy condition, but in contrast, since the stranger male is present in both conditions with the titi monkeys, the decrease in the right amygdala could be due to the positive presence of the pair-bond. This hypothesis is empirically supported, as titi monkeys did not display more aggressive behaviors (in contrast to the aggression demonstrated by macaques), but rather higher levels of lip smacking, an affiliative behavior probably directed to reinforcement of the pair bond. To test this, one might consider the inclusion of an additional control in future studies, i.e., the presentation of the female partner alone as for titi monkeys. However, as explained by the authors of both articles, for time, technical and financial reasons, all controls could not be tested with the same individuals. While higher plasma cortisol and testosterone in jealousy condition advocate in the favor of a jealous reaction, titi monkeys do not seem to react in an aggressive manner during jealousy. Rather, the activation of a specific brain area, the LS, an area rich in oxytocin, vasopressin and dopaminergic receptors in titi monkeys (Freeman et al., 2014b; Hostetler et al., 2017), suggests that mechanisms reinforcing bonding are activated during this challenging condition for this species.

One evolutionary interpretation of these findings in animals and humans would be that jealousy motivates an individual to try to “save” the existing relationship with their pair mate from disruption (Panksepp, 2010; Maninger et al., 2017b), but the behaviors that are involved are species-dependent. The sex differences shown in humans also clearly highlight the importance of studying both sexes when possible. Taken together, these studies demonstrate some similarities and some variation across species (Table 4), that might be partially related to the changes in experimental designs that need to be adapted to the species. These studies on jealousy are also a reminder that each emotion involves neural systems rather than one specific region.

### Separation from a valuable relationship: Social pain, loneliness, and grief

#### A word on emotional loneliness and social loneliness

In humans, social separation or loss are associated with emotions like loneliness and social pain (Eisenberger, 2006), but both terms are at times used interchangeably. Emotional loneliness in humans is typically assessed by questionnaires (for example the UCLA-LS, Russell et al., 1978) and sometimes with the use of the *Cyberball* game according to a recent review on the neurobiology of loneliness (Lam et al., 2021). Because these methods can only be used in humans, here, we focus primarily on studies of animals in which brain imaging was combined with scenarios like short-term and long-term social separation from valuable social partners, and we rely more on the term “separation distress.” Social separation is known to elicit depressive behavior in mice (Martin and Brown, 2010), and multiple NHP species (Worlein, 2014), so here, we also acknowledge that animals can potentially experience affective states akin to loneliness as well.

#### Social pain

Separation distress, or “social pain” is defined as a distressing experience due to the perception of a psychological distance and can be interpreted from an adaptive perspective as a process to promote social contact to strengthen relationships. For example, separation distress in maternal-infant bonds is viewed as a mechanism that elicits distress behaviors (e.g., calls) from the infant and maternal behavior from the mother, thus protecting the infant from estrangement and potential dangers of being alone (Eisenberger, 2006). Lesion studies in non-human mammals have first highlighted the role of the ACC, the anterior insula and the PAG in distress vocalizations (Eisenberger, 2012). Squirrel monkeys placed in isolation vocalized less when their ACC was ablated (MacLean and Newman, 1988). The electrical stimulation of the ACC produces vocalization in rhesus macaques (Smith, 1945). Rat pups separated from their mother show less distress call vocalization when the PAG is lesioned, and stimulation of the PAG can lead to spontaneous distress calls (Panksepp, 1998).

While the invasive studies cited above have pointed out the importance of the ACC in distress vocalization in pups and animal infants, the ACC has also been implicated in the neural response of the mother receiving the calls. Indeed, distress calls received by a mother separated from her infant elicit maternal reactions in animals and humans (Fleming and Rosenblatt, 1974; Ainsworth, 1978). As mentioned before (in the mother-infant attachment section) comparing the fMRI of mothers listening to their own infant cry or white noise revealed not only the activation of the ACC, but also other regions associated with maternal love and attachment (MPOA, VBNST, and VTA) (Lorberbaum et al., 1999, 2002).

Brain regions detected by fMRI for reactions related to social pain in a *Cyberball game* include the ACC and the right ventral prefrontal cortex (RVPFC) in humans (Eisenberger et al., 2003). An extensive body of work on human brain activation (reviewed in Eisenberger, 2012) advocates for a central role of the dorsal ACC and the anterior insula during social and physical pain, while the dorsal insula and the anterior insula are also activated when participants reported feeling of being excluded. The activation of the dorsal ACC is also modulated by parameters like friendship or self-esteem, which highlights how much physical pain and social pain rely on a similar neural basis (Eisenberger and Lieberman, 2004; Eisenberger, 2012). Interestingly, the dorsal ACC and the anterior insula which are activated during social pain, present a reduced activation when participants take pain killers such as acetaminophen (DeWall et al., 2010). However, there has been some criticism regarding the use of the *Cyberball* paradigm to study social rejection. One criticism is the hypothesis that any negative emotion or affective state may activate the ACC (*reviewed in* Eisenberger, 2015). Also, a recent meta-analysis on *Cyberball* studies found no evidence for the activation of the dorsal ACC during social exclusion (Mwilambwe-Tshilobo and Spreng, 2021), but that rejection engaged the default network instead.

One study on the topic of romantic rejection has employed an alternative paradigm in lieu of the *Cyberball* game, in which participants viewed a picture of themselves next to a second (fake) participant along with accompanying feedback from the second participant, noting if the second participant liked the real participant or not. Researchers identified an activation of the ventrolateral prefrontal cortex (vlPFC) and the anterior insula (Hsu et al., 2020), especially in men. However, this study also mentioned one limitation similar to the *Cyberball* game, which is the use of a fake participant as a stimulus. Studies with real participants as stimuli are rare (as reviewed in van der Watt et al., 2021), but these paradigms also generally engage the cingulate and the prefrontal cortex.

Until recently, little was known about the neural basis of separation anxiety in humans. An fMRI study in healthy human adults positively correlated *a priori* reports of higher levels of separation anxiety with a higher level of amygdalar activation in response to viewing negatively valanced faces (Redlich et al., 2015). However, this study did not directly image the neural response of anxiety during separation, but rather a correlation between an earlier, self-reported separation anxiety score during questionnaires and the later brain response during threatening face viewing. Authors also acknowledged that stimuli consisted of faces of strangers rather than those of close acquaintances; nevertheless, this study remains one of the rare examples of studies designed to determine the neural basis of separation anxiety in humans using fMRI. In another study, researchers compared healthy mothers to mothers with interpersonal violence-related posttraumatic stress disorder (PTSD) to assess neural activity while viewing their respective children in separation as compared to another child (Schechter et al., 2012). Mothers with PTSD showed decreased activation of the superior frontal gyrus and middle frontal gyrus. The comparison between the two studies is difficult given the two contexts (stranger threatening/neutral faces or own/stranger child) and given the differences in the methods (correlation of fMRI with anxiety score or direct analysis of fMRI), but they both converge in the sense that the amygdala and the superior frontal gyrus are implicated in negatively valenced stimulus (Schechter et al., 2012; Redlich et al., 2015).

#### Loneliness

Loneliness can be conceptualized as a form of social pain, and an ultimate, evolutionary origin for loneliness has been proposed (Cacioppo and Cacioppo, 2018). This recent evolutionary theory posits that loneliness is an adaptive mechanism that improves survivability of individuals found in a socially isolated situation by increasing motivation to look for social connection with other individuals and by increasing vigilance. According to this theory, neural mechanisms associated with motivational processes (including those that engage OT and dopamine) should be involved in loneliness as well as aversive responses (e.g., vigilance for social threats). A recent review highlighted the neural basis of loneliness in human subjects in studies using various structural/functional imaging and other non-imaging methods including computer tomography, MRI/fMRI, electroencephalography, diffusion tensor imaging, single-photon emission computed tomography, PET scans, and post- mortem brain tissue RNA expression or pathological analysis (Lam et al., 2021). Many of the studies on loneliness used the University of Los Angeles Loneliness Scale, a questionnaire designed to measure how much participants felt lonely. The collection of reviewed literature ultimately connected the feeling of loneliness with several brain areas, e.g., the PFC, the anterior insula, the amygdala, the hippocampus, and the posterior superior cortex. Interestingly, the PFC and the insula were also implicated in social pain, but there are relatively few mentions of the ACC found in studies on human social rejection in this particular review on loneliness.

#### Short-term separation in animals

The study of separation differs from that of loneliness in that it is a response to the loss of a specific individual. Short-term separations in young rhesus monkeys from their mother followed by a PET scan showed that the right dorsolateral PFC and the right ventral temporal/occipital lobe were both activated by maternal separation, and that these regional activations were positively correlated with cortisol level (possibly indicating a state of stress) (Rilling et al., 2001). Conversely, activity of the left dorsolateral PFC decreased with separation (Rilling et al., 2001). In titi monkeys (Hinde et al., 2016), researchers instead focused their investigation on the cingulate cortex because of *a priori* evidence from human studies of grief (Gündel et al., 2003) and social pain (Eisenberger, 2015); and authors also investigated regions in the reward system (i.e., the NAcc and VP), which were previously associated with grief in humans and prairie voles (O’Connor et al., 2008; Bosch et al., 2016). Researchers separated male titi monkeys from their female partner for 48 h and collected measures of neural activity from the males with PET imaging (Table 5). Following separation, imaging indicated decreases in glucose uptake in a number of regions (LS, VP, PAG, PVN of the hypothalamus, and cerebellum, Table 5) and an increase in CSF OT, plasma cortisol, and insulin (Hinde et al., 2016). The authors conclude that an increased release of OT and binding to OTR in the LS, and OT to AVPR1a in the PAG and cerebellum, could represent a potential mechanism of dealing with social separation and a preparation to the encounter of a new mate or an adaptive response to create a new bond quickly.

**TABLE 5:** Summary of the brain areas involved in the expression of separation across species.

Species (Sex)	How to elicit it (*Imaging method*)	Neural changes (↗ increase/↘ decrease)	References
Social pain and separation anxiety			
Humans–*Homo sapiens*	Participants playing *Cyberball* being excluded from a game *(fMRI)*	↗ **anterior cingulate cortex**, right ventral **prefrontal cortex**	Eisenberger et al., 2003
Humans–*Homo sapiens* *(Men and Women)*	Subjects receiving the feedback of another participant whether they like them or not along with their picture *(fMRI)*	*Romantic rejection* ↗ ventrolateral **prefrontal cortex** and anterior insula	Hsu et al., 2020 For a review: van der Watt et al., 2021
Humans–*Homo sapiens* *(Mothers)*	Mother listening to their own infant cry compared to white noise *(fMRI)*	↗ **anterior cingulate cortex**, medial preoptic area, medial preoptic area, bed nucleus of the stria terminalis	Lorberbaum et al., 1999, 2002
Humans–*Homo sapiens* *(Mothers)*	Mother (healthy group) viewing a separation from their children compared to another child *(fMRI)*	*Separation anxiety* ↘ medial **prefrontal cortex**	Schechter et al., 2012
Loneliness			
Humans–*Homo sapiens*	Social vs. non-social image presentation (functional imaging methods)	↗ **prefrontal cortex**, anterior insula, amygdala, hippocampus and posterior superior cortex	*Review paper*: Lam et al., 2021
Short-term loss			
Macaques–*Macaca mulatta* (*Males only*)	Separation of juveniles from their mother *(PET scan)*	↗ right dorsolateral **prefrontal cortex**, right ventral temporal/occipital lobe ↘ left dorsolateral **prefrontal cortex**	Rilling et al., 2001
Voles *–*Microtus ochrogaster* (*Males only*) *not a brain imaging study	Separation from the pair bond (3 days)	↘ of striatal oxytocin signaling in the nucleus accumbens shell	Bosch et al., 2016
Titi monkey–*Plecturocebus cupeus* *(Males only)*	Short-term separation from the pair bond (48 h) *(PET scan)*	↘ lateral septum, ventral pallidum, paraventricular nucleus of the hypothalamus, periaqueductal gray, and cerebellum	Hinde et al., 2016
Long-term loss and grief			
Titi monkey–*Plecturocebus cupeus* *(Males only)*	Long-term separation from the pair bond (2–3 weeks) *(PET scan)*	↘ reduced FDG uptake in the central amygdala, reduced whole brain FDG uptake	Hinde et al., 2016
Humans–*Homo sapiens* *(Women)*	Presentation of photographs or verbal evocation of a deceased *(fMRI)*	*By picture or verbal stimulation:* ↗ posterior cingulate cortex, medial/superior frontal gyrus, and cerebellum *Picture*: ↗ cuneus, superior lingual gyrus, **insula**, dorsal **anterior cingulate cortex**, inferior temporal gyrus, and fusiform gyrus *Verbal*: ↗ pre-cuneus, precentral gyrus, midbrain, and vermis	Gündel et al., 2003
Humans–*Homo sapiens* *(Women)*	Presentation of photographs or verbal evocation of a deceased *(fMRI)*	*Only for complicated grief:* ↗ nucleus accumbens *Normal and complicated grief:* ↗ **dorsal anterior cingulate cortex**, **insula**, periaqueductal central gray	O’Connor et al., 2008
Humans–*Homo sapiens* *(Women)*	Evocation of a pet loss within the last 3 months *(fMRI)*	↗ amygdala, the rostral and dorsal **anterior cingulate cortex**, as well as the dorsolateral PFC	Freed et al., 2009

Names in bold are brain part regularly found in neural changes related to emotion.

#### Long-term separation or loss

Bereavement or grief in humans may occur following a permanent loss and is and is expressed in various cultures (Eisenbruch, 1984). Studies of grief in humans present bereaved persons with visual stimuli depicting a deceased individual or alternatively with a verbal evocation. In women who had lost a first degree relative in the past year, PCC, medial/superior frontal gyrus, and cerebellum were activated by picture or verbal evocation of the deceased relative (Gündel et al., 2003). A variety of other regions were specifically increased by one stimulation or the other (see Table 5), and pet owners who have experienced loss of their animal also show activations of regions linked with sadness (Freed et al., 2009). Women who experienced the death of a mother or sister in the past 5 years and who have accordingly experienced complicated grief also show increased activation of the reward system (namely in the NAcc, O’Connor et al., 2008). These authors posit that this activation of the reward system may interfere with the process of adapting to the loss in the present.

Following a long-term (i.e., 2-week) period of social separation, PET imaging in male titi monkeys reflected a decrease in glucose uptake in the central amygdala and in the whole brain, as well as an increased CSF OT and increased plasma insulin concentrations (Hinde et al., 2016). This increase in CSF OT and insulin are interpreted as a sign of motivation for engaging in social interaction. The reduced, global glucose uptake of the brain associated with long-term separation seems to reflect a reverse process that contrasts with the increased, global glucose update which normally occurs during pair bond formation (Bales et al., 2007; Maninger et al., 2017a). Thus, there is mixed evidence: on one hand there appears to be activation of physiological processes meant to encourage social interaction; on the other hand, there are also processes at play that may reflect a process of adaptation to partner loss. In addition, titi monkey fathers that encountered a new stranger female showed lower glucose intake in the SON and the PVN, as well as a lower PCC uptake, when compared to non-fathers. When reunited with their pair-mates, fathers showed higher plasma cortisol concentrations, and lower CSF OT, plasma AVP and glucose concentrations than non-fathers. This higher PCC glucose uptake in non-fathers is interpreted as a potential higher openness/interest in a novel female, since the activation of the PCC has been implicated in the process of human partner choice (Yokoyama et al., 2017). This additional result reflects the need to consider the effects of other social factors and relationships that can modulate the response in brain activations between subjects as mentioned before in humans, but also that the emotions elicited by the loss of a bond might be mixed with the will or adaptation to create a new bond.

Some regions are regularly found to be activated by social pain (unrelated to death) in humans, which can be elicited, for example, by separation, loss or exclusion from a group (Eisenberger, 2006), namely the dorsal and ventral ACC and the anterior insula (as extensively reviewed by Rotge et al., 2015) and the rostro-ventral PFC (see Table 5). It is still complex to disentangle social pain, grief and separation in animals; however, the comparison between human and non-human species reveals striking similarities, therefore suggesting common evolutionary roots.

## Discussion, limitations, and further research

**FIGURE 1:**
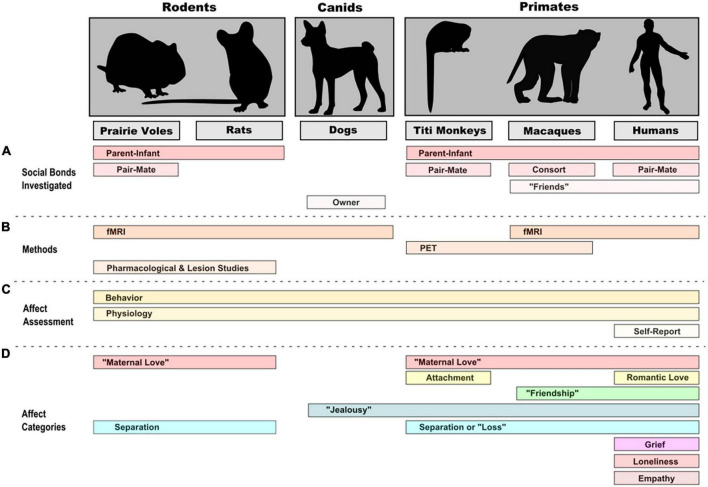
Social contexts **(A)**, methods **(B)**, and levels of assessment **(C)** in neuroimaging and other complementary studies of emotion/affect **(D)** in representative rodent, canid, and primate species.

Are there commonalities *between species* in the neural basis of social emotions? For that matter, are there neural commonalities *between emotions* that are related to being social? While it has been proposed (and we have discussed it here) that certain emotions are specifically social, there are a number of different perspectives on this issue. At least one empirical study failed to find a specifically social, fundamental dimension for emotion (Bliss-Moreau et al., 2020). Brain imaging studies of social emotion in animals, while still in their early days, have already provided critical insights into similarities and variation between species, studying a variety of social bonds and affective states with adapted imaging and emotion assessment methods (Figure 1), and have highlighted the implication of several brain areas (Table 6). Several considerations arise when considering the role that imaging can play in future studies on social emotion and the understanding of its evolution.

**TABLE 6:** Summary by brain areas of the regions most commonly involved in the expression the different social emotions across species.

Brain area	Neural changes in socioemotional contexts (↗ increase/↘ decrease)	Other associations–including non-imaging studies
ACC	*Romantic Love*: **↗** in humans (Bartels and Zeki, 2000) *Social and motivational reward*: **↗** in macaques (Chang et al., 2013) *Jealousy*: in **↗** in male titi monkeys (Maninger et al., 2017b) (left ACC) and women (Steis et al., 2021) *Grief*: **↗** in women (Gündel et al., 2003) and *Social Pain*: in **↘** humans (Eisenberger et al., 2003)	*Distress vocalizations* (Smith, 1945; MacLean and Newman, 1988; Eisenberger, 2012) *Empathy-Consolation* (Burkett et al., 2016)
Amygdala	*Romantic Love*: **↘** in humans (Bartels and Zeki, 2000; Acevedo et al., 2012) at early stages but also **↗** in the longer term *Long-term Bonding*: **↘** in male titi monkeys (Bales et al., 2007) *Jealousy:* **↗** in male macaques, dogs and men; **↘** in male titi monkeys (Rilling et al., 2004; Takahashi et al., 2006; Maninger et al., 2017b; Cook et al., 2018) *Lonelines*s: **↗** in humans (Lam et al., 2021)	*Fear* (Nesse, 1990) *Untrustworthiness of others* (Winston et al., 2002)
BNST		*Mother infant attachment* (Insel and Young, 2001)
Globus pallidus	*Jealousy*: ↗ in women (Steis et al., 2021)	
Hippocampus	*Romantic love and Feeling of friendship*: **↗** in humans (Bartels and Zeki, 2004; Güroğlu et al., 2008; Acevedo et al., 2012) *Lonelines*s: **↗** in humans (Lam et al., 2021)	
Insula	*Romanic and maternal love:* **↗** in humans (Bartels and Zeki, 2000, 2004) (medial insula) *Feeling of Friendship*: **↗** in humans (Acevedo et al., 2012) *Jealousy:* **↗** in male macaques, men and women (Rilling et al., 2004; Takahashi et al., 2006; Steis et al., 2021) (bilateral) *Romantic rejection and acceptance*: **↗** in humans (Hsu et al., 2020; van der Watt et al., 2021) *Grief:* **↗** in women (Gündel et al., 2003; O’Connor et al., 2008) and Social Pain: **↗** in humans (Eisenberger, 2012; Rotge et al., 2015) (anterior insula) and *Lonelines*s: **↗** in humans (Lam et al., 2021)	*Distress vocalizations* (Eisenberger, 2012)
LS	*Short-term Pair-bonding*: **↗** lateral septum (D1R binding) (Hostetler et al., 2017) and *Long-term Pair-bonding*: **↘** in male titi monkeys (Bales et al., 2007) *Jealousy*: **↗** in male titi monkeys (right LS) (Maninger et al., 2017b) *Separation*: **↗** in male titi monkeys (Hinde et al., 2016)	
MPOA		*Mother infant attachment* (Insel and Young, 2001)
NAcc	Romantic Love*: **↗** in humans (Acevedo et al., 2012), but **↘** in human in medial accumbens (Xu et al., 2011, 2012) and in titi monkeys in long-term bonds (Bales et al., 2007) *Conflicting directions *Feeling of Friendship*: **↗** in humans (Acevedo et al., 2012) *Grief*: **↗** in women (O’Connor et al., 2008)	*Separation* In voles: ↘ of striatal oxytocin signaling in the nucleus accumbens shell (Bosch et al., 2016)
OFC	Romantic love*: **↗** in human (Acevedo et al., 2012) and in the mid-OFC (Xu et al., 2011) and **↘** in human in medial OFC and negative correlation with happiness (Xu et al., 2011, 2012) *Conflicting directions *Maternal Love*: **↗** in human (Bartels and Zeki, 2004; Noriuchi et al., 2008) *Feeling of Friendship*: **↗** in human (Acevedo et al., 2012) and macaques (Azzi et al., 2012; Watson and Platt, 2012; Brent et al., 2014a)	*Untrustworthiness of others* (Winston et al., 2002)
PAG	*Maternal Love*: in humans (Bartels and Zeki, 2004; Noriuchi et al., 2008) *Romantic Love* and *Feeling of Friendship*: in humans (Acevedo et al., 2012)	*Maternal behaviors* (Miranda-Paiva et al., 2003) *Distress vocalizations* (Panksepp, 1998; Eisenberger, 2012)
PCC	*Romantic Love*: in humans (Bartels and Zeki, 2000) *Jealousy*: in ↗ male titi monkeys (Maninger et al., 2017b) and women (Steis et al., 2021) *Grief*: in women (Gündel et al., 2003)	*Happiness, Fear, sadness, and other unpleasant stimuli* (Maddock, 1999) *Partner choice* (Maninger et al., 2017a; Yokoyama et al., 2017)
PFC	*Feeling of Friendship:* **↗** in humans (Güroğlu et al., 2008)–ventro-medial PFC *Jealousy*: ↗ in women (Steis et al., 2021)–medial PFC *Separation*: ↗ in young macaques in the right dorsolateral PFC and **↘** in the left dorsolateral PFC (Rilling et al., 2001) and *Social Pain*: **↗** in humans (Eisenberger et al., 2003; Rotge et al., 2015) (rostro ventral PFC) *Romantic rejection and acceptance*: **↗** in humans (Hsu et al., 2020; van der Watt et al., 2021) and *Lonelines*s: **↗** in humans (Lam et al., 2021)	
PVN	*Separation (short-term)*: ↗ in male titi monkeys (Hinde et al., 2016)	
PCL	*Love:* ↗ in humans (Acevedo et al., 2012)	
STS	*Jealousy*: **↗** in male macaques (right) and women (posterior) (Rilling et al., 2004; Takahashi et al., 2006)	*Untrustworthiness of others* (Winston et al., 2002) *Gaze following in macaques* (Roy et al., 2014)
VP	*Attachment* ↘ in titi monkey pair bonding (Bales et al., 2007)	
VTA	*Romantic Love*: ↗ in humans (Bartels and Zeki, 2004; Fisher et al., 2005; Acevedo et al., 2012)	*Onset of maternal behavior* (Pedersen et al., 1994; Numan and Young, 2016) *Mother infant attachment* (Insel and Young, 2001)
SN	*Love:* ↗ in humans (Bartels and Zeki., 2004; Acevedo et al., 2012) *Jealousy*: ↗ in women (Steis et al., 2021)	

*Emphasize results going in conflicting directions.

### Unifying mechanisms for social emotions

One unifying mechanism for social emotions may be the actions of the neurohormone OT across mammalian species and across emotions, particularly in the instances of love or social separation, although vasopressin is also likely to play a role (Panksepp, 2010; Carter, 2014, 2017). The social salience hypothesis of OT (Shamay-Tsoory and Abu-Akel, 2016) suggests that OT’s focuses attention on social cues and social contexts, and OT mediates responses through interactions with the dopaminergic system. Neural systems involved in the production of or response to OT and dopamine thus make a reasonable place to look for common neural bases of emotion, and many of the studies cited here have done so.

One complication is that for many NHP species, the location of OT receptors is still not known (Freeman and Young, 2016). In fact, distributions have been published only for common marmosets (Schorscher-Petcu et al., 2009), rhesus monkeys (Freeman et al., 2014a), titi monkeys (Freeman et al., 2014b), and recently, several species of lemurs (Grebe et al., 2021) and chimpanzees (Rogers Flattery et al., 2021). There is relatively little overlap in these receptors between NHP species, although the overlap is much higher when the closely related vasopressin system is also considered (Freeman and Young, 2016). Attention should be paid to our basic knowledge of the OT system in each species, when considering what neural changes tell us about the neurobiology of emotion in that species.

### Evolutionary perspectives relative to social system

Non-human primates not only share a close evolutionary history with humans, but also present a large variety of social systems that could theoretically allow the study of how social system has impacted the evolution of emotions. However, the paucity of brain imaging studies in NHP allows us to draw few conclusions at this point. Monogamous titi monkeys showed similar engagement of brain structures as humans did during love, for instance, but showed some key differences during jealousy. Currently, it is often not possible to tell whether these distinct results are due to specificities of the social system, individual variation, or methodological differences. In future, many more species need to be studied, and experimental conditions should be matched as closely as possible to allow greater comparability between studies.

To aid in comparing studies, we recommend sharing protocols data on brain imaging of social emotions (Milham et al., 2020). Indeed, it would allow analyses with larger sample sizes and homogenize practice in the brain imaging analysis field that lack reproducibility (Poldrack et al., 2012). In primatology, researchers have proven their ability to work in a collaborative way, as for example in cognition with the ManyPrimates project (Altschul et al., 2019; ManyPrimates et al., 2019, 2020).

Primatologists focused on cognition have demonstrated a large effect of phylogeny over the effect of any ecological or social factor, at least for short-term memory, in an extremely large number of species (ManyPrimates et al., 2019; ManyPrimates, 2021). Given the tight relationship between cognition and emotion (Gosling, 2001; Kremer et al., 2020), one could reasonably hypothesize that the way affect and emotions are encoded in the brain is also highly constrained by phylogeny. This would mean that more closely related species have more comparable patterns of brain activations under the same condition than more distantly related species. This viewpoint is supported by the results on jealousy, in which jealous humans and macaques (more closely related) are more similar to one another than either are to more distantly related titi monkeys. An alternative (but not necessarily contradictory) perspective is that the social system of a species (e.g., monogamy or cooperative breeding) may promote similarity between phylogenetically distant species by means of convergent evolution. It is certainly possible for both explanations to be true in different situations.

Finally, we highlighted many non-human primate and mammal studies in this review, mainly because we were targeting brain-imaging studies, which were mainly conducted in these taxa. It is also important to keep in mind the importance of a larger range of taxa to be able to conduct a comparative analysis of social emotional responses to better understand its evolution. Indeed, vertebrate brains present a remarkable conservation of their Social Decision-Making Network (O’Connell and Hofmann, 2012), which could be important for social emotions like empathy (Tremblay et al., 2017). In addition, concerning the OT/AVP system and its relationship with the social system, a very important comparative work has been conducted in a subset of five finch species, all monogamous but who live in different group size: the comparative study of these example of species suggested that the evolution of the nonapeptide system could have been the main driver of social system convergence, leading to changes in group sizes in some species (Goodson and Kingsbury, 2011). Some evidence also suggest that, convergently with mammals, the role of the nonapeptides have the same function for offspring care in birds (Goodson et al., 2012).

### Neurochemical specificity

A significant, missing piece of information associated with measuring glucose uptake in PET scans is the identification of specific neurotransmitters involved in the activation of neurons within a specific brain region. However, it is also possible to use PET scans to explore these mechanisms more closely. As it has been proposed, PET scan studies can be conducted with specific radiotracers in order to target neurotransmitters that are circulating in the brain (Hostetler et al., 2017). For example, the use of a D1-receptor antagonist can detect the upregulation of D1-receptors in the brain after long-term pairing in titi monkeys (Hostetler et al., 2017). While radiotracers for some receptor systems (such as oxytocin and corticotropin-releasing hormone) remain elusive (Smith et al., 2016), tracers for other systems (dopamine, opioids, serotonin) have been used in humans and would mostly just require validation for the animal species being studied. In addition, pharmacological manipulation can be used in conjunction with imaging and behavior to give additional insight (Shamay-Tsoory and Abu-Akel, 2016).

### Lack of studies on females

A general caveat encountered in animal biology is the study bias toward males. This is especially the case in neuroscience in which many studies exclusively examine male subjects or participants. While this problem is less severe in human studies, 5.5 studies in males were reported for each study in females in animal neuroscience in year 2009 (Beery and Zucker, 2011). This disparity seems to have ameliorated over the past decade (Woitowich et al., 2020). Behavioral responses may at times be sexually dimorphic. For example, in monogamous prairie voles, females and males do not show the same patterns of neural or behavioral processes during pair bond formation and separation (Lieberwirth and Wang, 2016). In the case of jealousy, both studies that we reviewed on primates were conducted on males alone. One particular reason for that may be due to the fact that female monkeys may either demonstrate less jealousy-associated behavior, as is the case for titi monkeys (Cubicciotti and Mason, 1978); or alternatively, for female monkeys in a species that presents a polygynous social system, jealousy may be ecologically irrelevant (e.g., female macaques) (Thierry et al., 2004). However, given the complexities in attributing emotion in animals, the possibilities of feeling emotion without overt display of behavior, and the possibility of sex-specific neural substrates for similar emotions, studying these questions in both sexes should become standard.

### Choosing control conditions across studies

One challenge that becomes apparent in a review of the emotion and affect literature is the difficulty in choosing an appropriate or comparable control condition. Brain imaging is a relatively non-invasive procedure that allows one to test individuals in several tests and control conditions; however, brain imaging studies are very expensive, and it can prove financially burdensome or prohibitive to cultivate a large sample size (to capture variability) while simultaneously establishing several test conditions. Moreover, there are ethical limitations that preclude the imposition of too many stressful manipulations/experiments on any one individual. For this reason, researchers, in lieu of controlling for various conditions (e.g., alone or with the mate, with a stranger, or with a mate), instead generally select one relevant control condition. Clearly, more time and financial resources, as well as more coordinated studies, are needed to support brain imaging to standardize results in comparative studies on social emotion imaging in non-human animals. In general, brain imaging studies in animals acknowledge the difficulties encountered in choosing perfect control conditions as for the example in jealousy (Rilling et al., 2004; Maninger et al., 2017b). To cultivate conditions of jealousy or an appropriate control, the subject must perceive a threat to a valuable social relationship (Webb and de Waal, 2018) or the absence of that threat, respectively. These circumstances may be achieved by removing the presence of a competitor or, alternatively, by removing the valuable social stimulus. Ultimately, it is critical to establish a control condition that does not induce unwanted, extraneous social factors, for example additional stress or anxiety due to social isolation. Social isolation may be particularly important for some primate species, depending on their social system.

### All emotions are not investigated equally

While there is a relatively large body of work on romantic love and maternal love, especially in humans, there are many fewer brain imaging studies on other social emotions, for instance the feeling of friendship or jealousy, in either humans or animals. Jealousy studies in contexts other than consort or mate relationships, such as friendships or parent-infant relationships, are also very rare (Webb et al., 2020); even though jealousy is a social emotion that might be expressed very early by young children (Panksepp, 2010). Being able to target desired emotions in animals is difficult and relies on the identification of emotions that appear to be relevant for a given species, and accordingly, this research depends on creating appropriate stimulus situations that should evoke the desired emotion. For instance, how researchers should best study grief in animals remains challenging. We cannot assess the certainty of knowledge that an animal has about the loss of a social companion. We believe that these losses in animals that have attachment relationships could lead to a state like human grief. However, given our state of uncertainty we will refer to “loss” rather than “grief.” Other potential behavioral indicators of social emotion, such as “slow blink” in cats (Humphrey et al., 2020) or tail-wagging in dogs, could help us to access emotions like “trust” or perhaps even love in additional species.

Other social emotions associated with specific behaviors are regularly reported in primates and in pet dogs (Morris et al., 2008; de Waal, 2019), such as shame, pride (Tracy and Randles, 2011) or even the sense of fairness and inequity, and they represent an enormous and exciting field of investigation to explore using brain imaging technologies. Perhaps another complicated emotion related process to study would be empathy. Empathy has been described as our ability to be affected by the emotional state of another (de Waal, 2008), although other definitions have been argued. To our knowledge, neuroimaging studies on empathy or affect contagion have been published in humans but not in animals (Platek et al., 2005; Decety and Grèzes, 2006). If one is to acknowledge the presence of emotion or affect in animals, there is perhaps no reason to deny the presence of empathy or related processes in animals; and, subtle experimental designs are needed to image empathy and related processes (e.g., affect or stress contagion). For example, which regions of the brain are activated when a macaque or a chimpanzee observes a conspecific yawning (Anderson et al., 2004; Paukner and Anderson, 2006)? Do patterns of neural activation of dogs reflect the process of affect contagion or empathy for other dogs?

In humans, empathy of social pain has been imaged in humans looking at other people being socially excluded, with highly empathic people also exhibiting patterns of brain activation associated with social pain (anterior insula and dorsal ACC) (Masten et al., 2011). Yet, only a few experiments have been attempted involving animals (and not always mentioning empathy *per se*). Rodent species can show helping behaviors and consolation behavior, so they also seem to be good models for the study of the physiology and neurological basis of empathy and have helped to describe the implication of oxytocin and of the ACC in this emotion related process (Burkett et al., 2016; Cox and Reichel, 2021; Mason, 2021). A human-animal fMRI study has been conducted with humans attributing emotions to humans and to animals (Spunt et al., 2017); the same neural mechanisms were involved in the attribution of emotions in humans and animals, involving dorsomedial and lateral orbitofrontal prefrontal cortices. Such experiments could be further investigated in animals.

## Conclusion

Because of functional neuroimaging, researchers have been able to compare neural activation not only in specific areas, but also brain networks linked to similar emotional situations in humans and animals. This has allowed researchers to suggest with more certainty that the affective lives of animals are often shaped by the same or similar neural substrates, and perhaps result in the same or similar subjective affective experiences, as do those of humans. Common social scenarios often generate similar patterns of neural activation between humans and NHP; for example, the engagement of dopamine rich regions during positive emotions like love and attachment, and the involvement of the amygdala for emotions associated with a variation in arousal. However, one also finds significant inter-species variation, as well as intra-species variation reflective of sex differences. In addition, the OT system is likely to be of importance for social emotion across species. In the titi monkey, the LS (which contains both OT and dopamine receptors) shows activation or deactivation in each emotion considered in this review. The differences between animals and humans are well illustrated by titi monkeys, but also put into context by our closer relatives, rhesus macaques. Of particular importance may be the localization and density of OT and dopamine receptors, particularly in the studies of love and attachment. The studies presented here illustrate how brain imaging can help researchers investigate the neural basis of complex affective states and emotions. Conducting brain imaging on more species in the future will help to disentangle ecological vs. phylogenetic effects.

## Author contributions

PZ-T drafted the manuscript. PZ-T, FR, and KB participated in the writing and reviewed the manuscript. KB provided financial support. All authors contributed to the article and approved the submitted version.
